# Comparison of a 20 degree and 70 degree tilt test in adolescent myalgic encephalomyelitis/chronic fatigue syndrome (ME/CFS) patients

**DOI:** 10.3389/fped.2023.1169447

**Published:** 2023-05-12

**Authors:** C. (Linda) M. C. van Campen, Peter C. Rowe, Frans C. Visser

**Affiliations:** ^1^ Stichting CardioZorg, Hoofddorp, Netherlands; ^2^ Department of Pediatrics, Johns Hopkins University School of Medicine, Baltimore, MD, United States

**Keywords:** orthostatic intolerance, cerebral blood flow, tilt-table testing, myalgic encephalomyelitis, adolescents, 20 degree, 70 degree

## Abstract

**Introduction:**

During a standard 70-degree head-up tilt test, 90% of adults with myalgic encephalomyelitis/chronic fatigue syndrome (ME/CFS) develop an abnormal reduction in cerebral blood flow (CBF). A 70-degree test might not be tolerated by young ME/CFS patients because of the high incidence of syncopal spells. This study examined whether a test at 20 degrees would be sufficient to provoke important reductions in CBF in young ME/CFS patients.

**Methods:**

We analyzed 83 studies of adolescent ME/CFS patients. We assessed CBF using extracranial Doppler measurements of the internal carotid and vertebral arteries supine and during the tilt. We studied 42 adolescents during a 20 degree and 41 during a 70 degree test.

**Results:**

At 20 degrees, no patients developed postural orthostatic tachycardia (POTS), compared to 32% at 70 degrees (*p* = 0.0002). The CBF reduction during the 20 degree tilt of −27(6)% was slightly less than during the reduction during a 70 degree test [−31(7)%; *p* = 0.003]. Seventeen adolescents had CBF measurements at both 20 and 70 degrees. The CBF reduction in these patients with both a 20 and 70 degrees test was significantly larger at 70 degrees than at 20 degrees (*p* < 0.0001).

**Conclusions:**

A 20 degree tilt in young ME/CFS patients resulted in a CBF reduction comparable to that in adult patients during a 70 degree test. The lower tilt angle provoked less POTS, emphasizing the importance of using the 70 degree angle for that diagnosis. Further study is needed to explore whether CBF measurements during tilt provide an improved standard for classifying orthostatic intolerance.

## Introduction

Adult and pediatric myalgic encephalomyelitis/chronic fatigue syndrome (ME/CFS) patients have a high prevalence of orthostatic intolerance (1–5). In response to the high rate of false positive syncope of up to 40% of young individuals during tilt-testing (6, 7), some investigators have employed a less severe orthostatic stress in adolescents with ME/CFS (7). A tilt angle of just 20 degrees was capable of provoking significant differences between ME/CFS patients and healthy controls, notably with a significantly larger decrease in stroke volume in ME/CFS participants (7). This finding is consistent with the hypothesis that a reduction in venous return and thus a reduction in stroke volume is one of the most important mechanisms of orthostatic intolerance.

In a study using extracranial Doppler measurements of the internal carotid and vertebral arteries, we recently demonstrated that 90% of 429 adult ME/CFS patients had an abnormal cerebral blood flow (CBF) reduction during a 70-degree tilt for up to 30 min (5). This abnormal CBF reduction was not only present in ME/CFS patients with well-defined heart rate and blood pressure abnormalities during tilt testing, like orthostatic hypotension, postural orthostatic tachycardia syndrome (POTS), and syncope, but also in ME/CFS patients with a normal heart rate and blood pressure response to upright posture. The mean CBF reduction of 26% in the entire study population with ME/CFS was significantly different from the 7% reduction observed in healthy controls.

We previously used a less severe tilt angle of 20 degrees to evaluate CBF in 19 adults with severe ME/CFS who we judged to be unable to tolerate the 70 degree angle. Their mean CBF reduction at 20 degrees was 27%, confirming that CBF abnormalities are already identifiable in patients with severe ME/CFS at the lesser tilt angle (8).

Given the high false positive syncopal rates among adolescents during a more severe orthostatic stress, the observation that a 20 degree tilt angle could provoke a reduced stroke volume in adolescents with ME/CFS, and the potential for less provocation of post-test symptoms in affected patients, a 20 degree tilt angle might be an acceptable alternative to the standard 70 degree angle. We compared CBF measurements and hemodynamic results at 20 degree and 70 degree tilt angles in adolescents with ME/CFS. Our objective was to determine whether the 20 degree angle would provide adequate diagnostic sensitivity for hemodynamic abnormalities like POTS, or for clinically significant changes in CBF.

## Methods

### Patients

From November 2015 to October 2022, we evaluated consecutive patients aged 10–18 years who were referred to the outpatient clinic of the Stichting CardioZorg, Hoofddorp, the Netherlands, because of the suspicion of ME/CFS and with symptoms of orthostatic intolerance in daily life. All patients underwent tilt-testing. Based on the clinical history, patients were classified as having CFS, chronic fatigue, or no chronic fatigue as defined by Fukuda and colleagues (9) and as having ME or no ME as defined by Carruthers and colleagues (10), taking the exclusion criteria of both case definitions into account. In none of the patients was another disease or biochemical abnormality available to explain the symptomatology. From November of 2015 until June 2019 we tilted the adolescents at the standard 70 degrees. Under the assumption that a lower orthostatic stress reduces the orthostatic stress burden and likelihood of post-exertional malaise, we shifted to a low grade orthostatic stress protocol of 20 degrees tilting from July 2019 until the present time. The tilt duration varied between 10 and 15 min. If a specific request in the referral was made to diagnose POTS, patients were tilted initially at a low grade (20 degrees) orthostatic stress, followed by the standard (70 degrees) orthostatic stress in one session. In this double tilt session, the 70 degree tilt lasted between 5 and 10 min. If patients developed severe orthostatic symptoms the test was stopped prematurely, after obtaining the upright images. None of the studied adolescents used heart rate and blood pressure lowering medication.

To classify ME/CFS severity, we used the International Consensus Criteria (ICC). Mild severity required an approximate 50% reduction in pre-illness activity level. Moderate severity required patients to be mostly housebound. Severe patients were mostly bedridden and very severe patients were totally bedridden and needed help with basic functions (10). Very severe patients were not studied because they were unable to tolerate tilt testing.

The study was carried out in accordance with the Declaration of Helsinki. All ME/CFS patients and/or their parents/caretakers gave informed, written consent. The study was approved by the medical ethics committee of the Slotervaart Hospital, Amsterdam, the Netherlands (reference number P1736).

### Head-up tilt test with cerebral blood flow measurements

Measurements were performed as described previously (8, 11). Heart rate, systolic, and diastolic blood pressures were continuously recorded by finger plethysmography using the Nexfin device (BMeye, Amsterdam, the Netherlands). Heart rate and blood pressures were extracted from the device and imported into an Excel spreadsheet. Bilateral internal carotid artery and vertebral artery Doppler flow velocity frames were acquired, using a Vivid-I system (GE Healthcare, Hoevelaken, the Netherlands) equipped with a 6–13 MHz linear transducer. High resolution B mode images, color Doppler images and the Doppler velocity spectrum (pulsed wave mode) were recorded in one frame. At least two consecutive series of six frames per artery were recorded. End-tidal PCO_2_ (P_ET_CO_2_) was monitored using a Lifesense device (Nonin Medical, Minneapolis USA).

### Data analysis

The changes in heart rate and blood pressure during head-up tilt test were classified according to consensus guidelines (12, 13): normal heart rate and blood pressure response, classic orthostatic hypotension, delayed orthostatic hypotension, POTS, and syncope or near-syncope. According to the guidelines POTS was defined by an increase in heart rate >= 40 bpm in adolescents.

Blood flows of the internal carotid and vertebral arteries were calculated offline by an investigator (CMCvC) who was unaware of the patient severity status. Blood flow in each vessel was calculated from the mean blood flow velocities times the corrected cross-sectional area (14, 15) and expressed in ml/min. Flow in the individual arteries was calculated in 3–6 cardiac cycles and data were averaged. Total CBF was calculated by adding the flow of the four arteries. We previously demonstrated that this methodology had good intra- and inter-observer variability (11).

### Statistical analysis

Data were analyzed using Graphpad Prism version 6.05 (Graphpad software, La Jolla, California, USA). All continuous data were tested for normal distribution using the D'Agostino-Pearson omnibus normality test, and presented as mean (SD) or as median with the IQR, where appropriate. For continuous data paired or non-paired t-tests were used for comparison, where appropriate. A *p* value of <0.05 was considered significant.

## Results

### Participants

**Figure 1:**
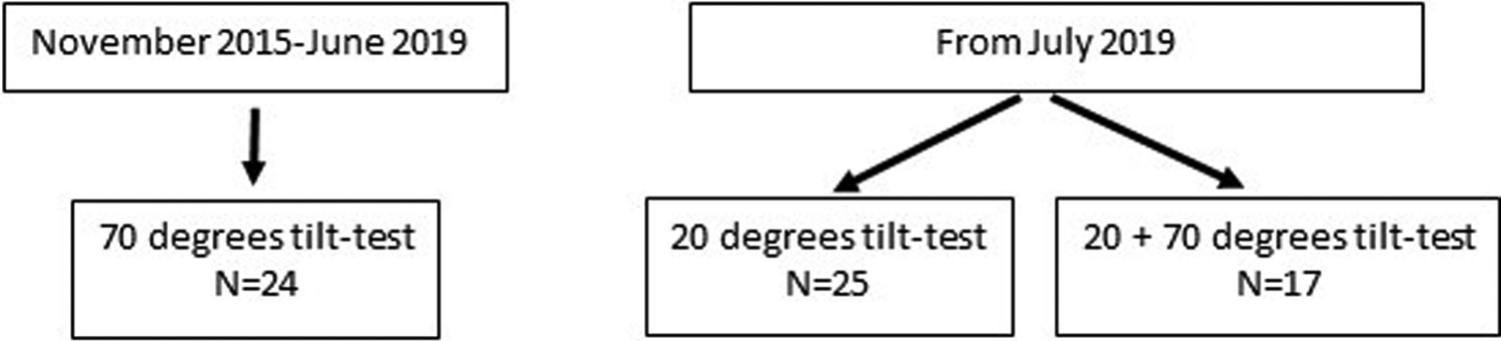
Patient flow.

We evaluated 24 adolescents between November 2015 and June 2019 using a 70 degree tilt angle. After June 2019, we studied 42 at a 20 degree angle, 17 of whom had the double tilt study at both 20 and 70 degrees. This resulted in 41 studies at 70 degrees, and 42 at a 20 degree angle. Figure 1 shows the patient flow. The demographic data of the 17 patients with both a 20 degree tilt and a 70 degree tilt in one session were compared with the data of the 25 patients with a 20-degree tilt, and with the 24 patients with a 70-degree tilt. None of the demographic results were significantly different in the comparison of either the two 20 degree groups or the two 70 degree groups. Therefore, the 20 degree studies of the 17 patients with both a 20 and 70 degree test were combined with the 25 patients with only a 20 degree test. In a similar manner the 70 degree data of the 17 patients with a double test were combined with 24 patients with a single 70 degree test. The test was stopped prematurely in 6 patients. All 66 patients met the Fukuda criteria for CFS, and all met the ICC criteria for ME. Daily-life orthostatic intolerance symptoms were reported by all ME/CFS adolescent patients. Baseline characteristics are shown in Table 1. The group tilted to 20 degrees was slightly younger: 16 (14.8–17.0) vs. 17 (15.5–18.0) years (*p* = 0.007), weighed less: 57 (14) vs. 64 (16) kg (*p* = 0.05), and had lower BMI: 20.1 (3.8) vs. 22.1 (4.9) kg/m^2^ (*p* = 0.04). No differences were found in disease duration or disease severity between the groups.

**Table 1:** Baseline data of adolescent ME/CFS patients with a 20 degree tilt test compared to adolescent ME/CFS patients with a 70 degree tilt test.

	20° tilt group (*n* = 42)	70° tilt group (*n* = 41)	*p*-value
Male/female^a^	14/28 (33/67%)	10/31 (24/76%)	0.37
Age (years)^b^	16 (14.8–17.0)	17 (15.5–18.0)	0.007
Height (cm)	168 (10)	170 (9)	0.51
Weight (kg)	57 (14)	64 (16)	0.05
BSA (m^2^)	1.65 (0.20)	1.73 (0.22)	0.08
BMI (kg/m^2^)	20.1 (3.8)	22.1 (4.9)	0.04
Disease duration (years)^b^	3.5 (2–7)	5 (2–7)	0.71
Disease severity^a^	13/20/9 (31/48/21%)	14/21/6 (34/51/15%)	0.72

^a^ Chi-square analysis (2 × 2 or 2 × 3 table); Disease severity x/y/z: x = mild disease, y = moderate disease, z = severe disease.

^b^ Median (IQR) compared by Mann-Whitney test; BSA, body surface area (duBois formula); BMI, body mass index.

### Comparison of 20 and 70 degree tilt angles

Table 2 shows the comparison of the hemodynamic results of orthostatic stress testing between the 20 degree tilt and the 70 degree tilt studies in ME/CFS adolescent patients. The group tilted at 70 degrees had a higher prevalence of POTS: 13 of 41 (32%) vs. 0 of 42 (0%), *p* = 0.0002. As a consequence of a higher percent of patients with POTS, the heart rate at end-tilt was significantly higher in the 70 degree group than in the 20 degree group: 103 (21) vs. 83 (15) bpm (*p* < 0.0001). No differences in blood pressures were documented. Orthostatic hypotension occurred in 3 patients with only one tilt degree angle: one at a single 20 degree study and two at the single 70 degree study. In one patient with both a 20 and 70 degree test the orthostatic hypotension was established during the post-hoc analysis of the heart rate and blood pressure data. Therefore, in the hemodynamic analysis, the orthostatic hypotension of this patient was added also to the data of the one patient with only a 20 degree study and to the two patients with only a 70 degree study.

**Table 2:** Hemodynamic data of adolescent ME/CFS patients with a 20 degree tilt test compared to adolescent ME/CFS patients with a 70 degree tilt test.

	20° tilt group (*n* = 42)	70° tilt group (*n* = 41)	*p*-value
Heart rate supine (bpm)	71 (12)	74 (14)	0.35
Heart rate end-tilt (bpm)	83 (15)	103 (21)	<0.0001
SBP supine (mmHg)	117 (17)	121 (18)	0.19
SBP end-tilt (mmHg)	115 (15)	115 (14)	0.96
DBP supine (mmHg)	72 (15)	76 (15)	0.30
DBP end-tilt (mmHg)	75 (13)	80 (12)	0.10
P_ET_CO_2_ supine (mmHg)	39 (3)	38 (3)	0.34
P_ET_CO_2_ end-tilt (mmHg)	33 (4)	30 (5)	0.02
CBF supine (ml/min)	702 (96)	712 (116)	0.68
CBF end-tilt (ml/min)	516 (90)	490 (90)	0.19
% CBF reduction (%)	−27 (6)	−31 (7)	0.005

Hemodynamic tilt test results: HR, heart rate; SBP, systolic blood pressure; DBP, diastolic blood pressure; P_ET_CO_2_: end-tidal CO2; CBF, cerebral blood flow.

**Figure 2:**
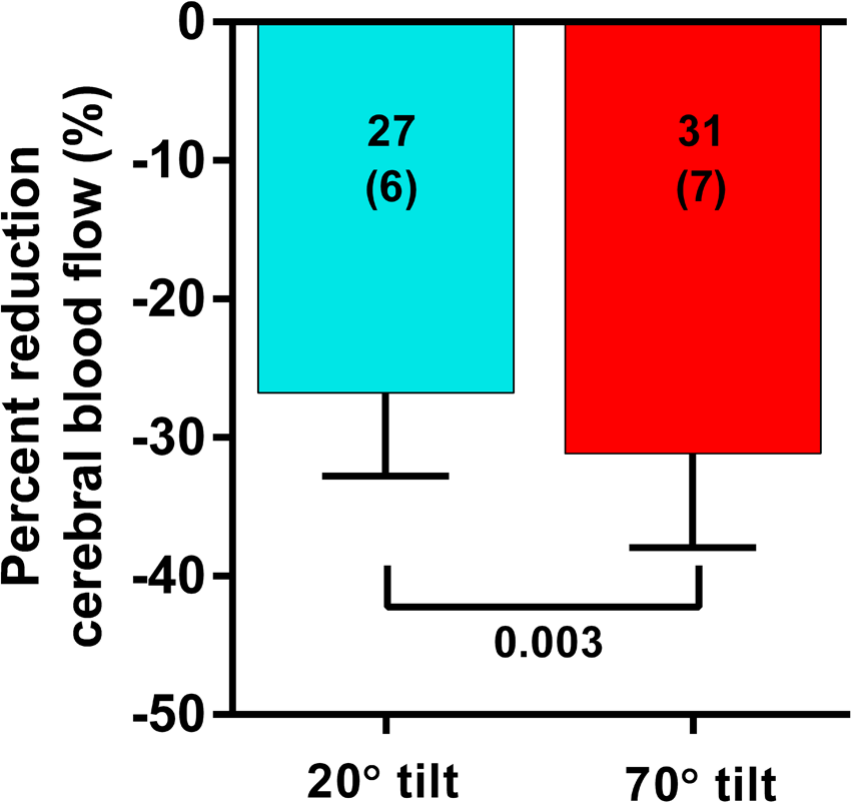
Percent reduction of cerebral blood flow in patients undergoing a 20 degree or a 70 degree tilt test. Legend figure 2: %CBF: percent reduction in cerebral blood flow between supine and end-tilt.

Although the CBF supine and end-tilt were not significantly different between the 20 degree and the 70 degree studies, the percent reduction in CBF at end-tilt was larger in the adolescents undergoing a 70 degree tilt: −31 (7)% in 70 degree studies vs. −27 (6)% in the 20 degree studies (*p* = 0.005). Figure 2 is a graphical representation of the percent CBF reduction. At end-tilt, end-tidal CO2 was slightly lower in 70 degree studies: 30 (5) vs. 33 (4); *p* = 0.02. No near-syncope or syncope was observed at 20 degrees, compared to 3 at 70 degrees; these patients were tilted back immediately after onset of the near-syncopal complaints. In these three patients the near-syncopal episodes occurred after the upright Doppler flow images had been acquired, allowing inclusion in the present study. The CBF reduction in these three patients was similar to the reduction in the remaining 37 patients: −29 (5)% vs. −31 (7)% (*p* = 0.56).

### Comparison of combined 20 and 70 degree tilt angle studies

**Figure 3:**
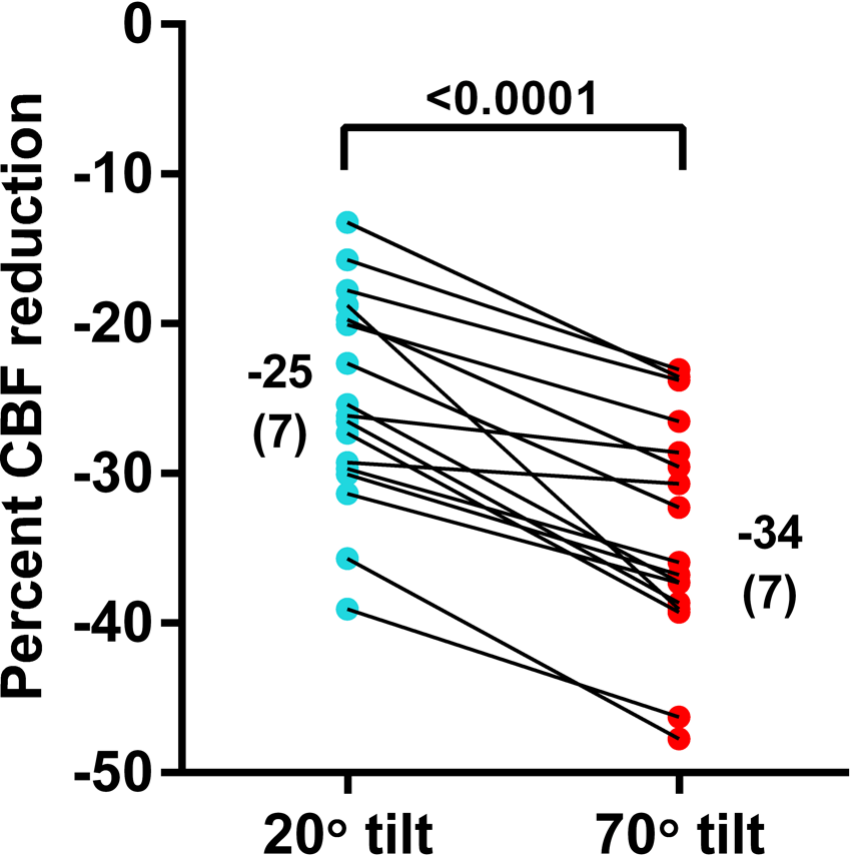
Percent reduction of cerebral blood flow in patients undergoing a 20 degrees tilt, followed by a 70 degree tilt. Legend figure 3: %CBF reduction: percent reduction in cerebral blood flow between supine and end-tilt.

In the 17 adolescents (5 males and 12 females) who underwent both 20 degree and 70 degree measurements during one session, the median age was 17 (15.4–17.1) years; 8 adolescents had mild disease, 6 had moderate disease, 3 had severe disease. The distribution of disease severity in groups with 20 degrees, 70 degrees, and combined 20–70 degrees tilting showed no significant difference (*p* = 0.276). Median disease duration was 4 (2–7) years. Table 3 shows the hemodynamic results of the paired analysis. The prevalence of POTS was significantly higher during the 70 degree tilt (*p* = 0.002). As a consequence of the higher prevalence of POTS in the 70 degree studies heart rate was significantly higher in this group (*p* = 0.003). No significant differences were found in blood pressures supine and end-tilt. A significant difference was observed in the end-tilt CBF: 529 (92) at 20 degrees and 467 (84) at 70 degrees (*p* < 0.0001). Figure 3 illustrates the percent reduction in CBF: −25 (7)% at 20 degrees vs. −34 (7)% at 70 degrees (*p* < 0.0001).

**Table 3:** Hemodynamic data of adolescent ME/CFS patients with both a 20 degree tilt test and a 70 degree tilt test.

*N* = 17	20° tilt	70° tilt	*p*-value
Hemodynamic results*	16/1/0 (94/6/0%)	7/2/8 (41/12/47%)	0.003
Heart rate supine (bpm)	71 (13)		1.0
Heart rate end-tilt (bpm)	86 (17)	96 (18)	0.003
SBP supine (mmHg)	123 (16)		1.0
SBP end-tilt (mmHg)	117 (12)	115 (14)	0.29
DBP supine (mmHg)	72 (12)		1.0
DBP end-tilt (mmHg)	77 (10)	77 (12)	0.71
P_ET_CO_2_ supine (mmHg)	40 (3)		1.0
P_ET_CO_2_ end-tilt (mmHg)	34 (5)	32 (4)	0.04
CBF supine (ml/min)	712 (97)		1.0
CBF end-tilt (ml/min)	528 (92)	467 (84)	<0.0001
% CBF reduction (%)	−25 (7)	−34 (7)	<0.0001

* Represents chi-square analysis.

Hemodynamic tilt-test results: x/y/z, x = normal heart rate and blood pressure response, y = orthostatic hypotension, z = postural orthostatic tachycardia syndrome; HR, heart rate; SBP, systolic blood pressure; DBP, diastolic blood pressure; P_ET_CO_2_: end-tidal CO2; CBF, cerebral blood flow.

## Discussion

The main findings of this study are that in adolescents with ME/CFS a large CBF reduction was observed even at a 20 degree tilt, and that hemodynamic criteria for POTS are less likely to be present in response to a 20 degree upright tilt than in response to a 70 degree test. While the lesser tilt angle provoked a lower heart rate response and a less impressive reduction in CBF, the 20 degree angle was capable of provoking a substantial reduction in CBF.

In a large, recent study we observed that adult ME/CFS patients had a significantly larger CBF reduction (mean −26%) compared to healthy controls (mean −7%) during a 30 min 70 degree head-up tilt test. In these ME/CFS patients significantly more orthostatic intolerance complaints were observed than in healthy controls, and there was a positive relation between the CBF reduction and the number of orthostatic intolerance complaints (5). We also performed a 20 degree tilt study in adult, severe ME/CFS patients, showing a similar CBF reduction of −27% as in milder diseased patients during a 70 degree tilt (8). In the present study the −27% CBF reduction after 10–15 min of a 20-degree tilt test compares with the two adult ME/CFS studies (5, 8). Therefore, although we did not include adolescent healthy controls, it is reasonable to assume that the CBF abnormalities in the adolescent ME/CFS patients are a true pathological finding as in the adult population.

Furthermore we observed a significantly lower CBF reduction at a 20 degree tilt than at a 70 degree tilt in the adolescents. The mechanisms of the cerebral flow regulation are complex and involve the cerebral perfusion pressure, PO2 and PCO2, flow-metabolism coupling, innervation of cerebral vessels, and blood viscosity (16). In ME/CFS patients an abnormally decreased venous return due to the orthostatic stress (17, 18), differences in blood volume (19, 20), leg venous distensibility (21, 22) muscle blood pump characteristics (23), deconditioning (24),sympathetic drive (25), the hemodynamic abnormality during a tilt test (5), chronotropic incompetence (26), inflammation (27), autoimmunity of the nervous system (28), endothelial dysfunction (29), disease severity (30) and microclots (31) may play a role. Nevertheless, our data suggest that the degree of orthostatic stress plays an important role in the CBF regulation. This influence is not a unique feature for ME/CFS as the influence of the degree of orthostatic stress has also been observed in healthy controls where the stroke volume reduction was dependent on the tilt test angle: higher tilt angles resulted in larger stroke volume reductions (32).

Also, our data suggest that the CBF reduction is larger in adolescents than in adults. In the present study the CBF reduction at 70 degrees was −31% whereas the CBF in adult ME/CFS patients was −26% (5). In a recent review Meng et al. showed that acute cardiac output changes result in CBF changes in the same direction (33). Previous studies showed that the cardiac output changes during a tilt test were, amongst others, dependent on age. Older patients showed less cardiac output reduction than younger patients. For example, Youde et al. studied healthy controls with a mean age of 69 years and found that cardiac output initially decreased but returned to supine values within 1 min (34). On the other hand Shoemaker et al. studied healthy young female controls with a mean age of 26 years and found a cardiac index decrease of 43% (35) during a tilt test. It is therefore conceivable that the CBF reductions in ME/CFS patients are related to cardiac output reductions with differences between the old and young. This needs to be studied in the future as well as whether these differences are clinically relevant. Furthermore, in the present study end-tilt P_ET_CO_2_ was slightly lower during the 70 degree tilt than during the 20 degree tilt test. The effect of lowering P_ET_CO_2_ (hypocapnia) on CBF reduction has been studied extensively in health and disease (16), and also in ME/CFS patients (36). It remains to be determined to what extent the PETCO2 reduction during the tilt contributes to the CBF reduction.

The lower orthostatic impact of the 20 degree is further exemplified by the absence of development of POTS, being significantly less compared to the 32% prevalence in the group with a 70 degree tilt. This was again confirmed in the direct comparison of the patient group who had both a 20 and 70 degree tilt test. POTS is a complex, multi-system and multifactorial disorder of the autonomic nervous system characterized by orthostatic intolerance with excessive heart rate increase and symptoms on standing while blood pressure is maintained (37). There are multiple possible mechanisms involved, like hypovolemia, deconditioning, inflammation, excessive sympathetic activation, auto-antibodies, small fiber neuropathy, and venous vessel laxity (37). Our data suggest that the degree of orthostatic stress plays a role by influencing the impact of the aforementioned mechanisms via circulatory (venous return), hormonal and cerebral flow abnormalities. When the diagnosis of POTS is important for patient management (diagnosis and treatment) the 20 degree tilt test, followed by a short 70 degree test can be used. Although the baseline data were not different between the groups, it is conceivable that the abovementioned mechanisms (hypovolemia, small fiber neuropathy, and vessel laxity) maybe different between the groups. A more comprehensive study involving these mechanisms is needed.

As observed in our previous study and other recent studies using transcranial Doppler we again demonstrated in these adolescent patients that orthostatic intolerance/abnormal CBF reductions can be present without heart rate and blood pressure abnormalities (5, 38–40). In our present study of patients undergoing both a 20 degree and a 70 degree tilt, 16/17 (94%) patients during the 20 degree tilt, and 7/17 (41%) patients during the 70 degree tilt showed a normal heart rate and blood pressure response. Patients with a normal heart rate and blood pressure response during a tilt test might be misclassified as having no abnormalities. The present study suggests that CBF measurements are needed in order to more accurately measure the prevalence of orthostatic intolerance in ME/CFS patients, especially in those with orthostatic intolerance symptoms. Our results of extracranial Doppler measurements are consistent with results in adolescents when using transcranial Doppler (36, 41).

We changed our previously described protocol (5) by reducing the tilt angle to 20 degrees and by shortening the duration upright (10–15 min vs. 30 min). In our previous publication (5) the mid-tilt CBF acquisition was started at 12 min, comparable to the acquisition time in the present study. In the former study the CBF abnormalities were slightly (but significantly) less at mid-tilt vs. end-tilt in all three patient groups studied with a normal heart rate and blood pressure, with orthostatic hypotension and with POTS. This pattern was also observed in healthy controls. Therefore, a larger decrease in CBF can be expected when a longer tilt duration is applied. Nevertheless, the differences between healthy controls and the patients were large and it is to be expected that the diagnostic yield would remain unchanged. However, this needs to be established in future studies. Another advantage of the lower tilt angle is that none of the adolescents experienced syncope or near-syncope at 20 degrees, compared to 1 syncope at 70 degrees and 2 near-syncopal episodes. Acquisition of Doppler images to calculate flow through both internal carotid and both vertebral arteries takes approximately 3 min. The milder orthostatic stress allows accurate measurement of CBF reductions that might have been difficult to measure in those who have rapid drops in blood pressure associated with classical orthostatic hypotension or vasovagal syncope when tested at 70 degrees (5). We had previously shown that tilt testing is associated with a prolonged increase in symptoms, evident at least one week after the orthostatic stress (42). The reduction in duration and the degree of orthostatic stress with a 20 degree tilt has the potential to reduce the orthostatic stress burden and the duration of post-exertional malaise. This hypothesis needs to be tested in future studies.

Our data, combined with the observation of high false-positive syncope rates in younger patients during a 70 degree tilt test suggest a diagnostic role for the lower tilt angle in pediatric ME/CFS patients. However, we caution that the 20 degree head-up tilt angle needs further study before it can replace the longer 70 degree tilt angles for assessing orthostatic intolerance in the pediatric ME/CFS patients.

### Limitations

This study only included consecutive adolescents with ME/CFS where the decision to tilt to 70 or 20 degrees was made based on the judgment of the treating clinician, and with a desire to reduce post-tilt symptoms. This may have resulted in an inclusion bias. Tilting at 20 degrees makes it less likely to evoke a POTS response. There is a significant difference in age between the 20 and 70 degree tilt groups, however the median difference is one year. Although younger patients are more prone to syncope, we are convinced that this one year difference is not clinically relevant. We did not perform a standard 20 degree, followed by a 70 degree tilt test in all patients, this may also have resulted in bias. However, the baseline profile of those who underwent a 20 plus 70 degree test was not different from those with either a 20 degree or a 70 degree test. We also did not include healthy young controls for comparison. A larger study, including healthy adolescents may give more insight into the pathophysiology. However, it is possible that healthy young controls would have little or no perturbation in response to a 20 degree head-up angle, which may have the effect of widening the (patho)physiological differences between ME/CFS patients and controls. Whether disease severity differences lead to differences in CBF reduction in adolescents, needs to be studied in the future. Finally, due to the relative low number of adolescents studied, a subgroup analysis of patients with a normal heart rate and blood pressure response, with orthostatic hypotension and with POTS was not feasible, but could be conducted in a larger group of adolescents.

## Conclusion

This study demonstrates that a short 10–15-minute tilt using a mild 20 degree head-up angle is less capable of provoking POTS than during a tilt to a 70 degree angle, but is sufficient to provoke a clinically substantial reduction in CBF in young patients 18 years or younger without inducing syncope. This method of orthostatic testing has the potential to improve the assessment of the prevalence of orthostatic intolerance in young affected ME/CFS patients who are reluctant to undergo a 70 degree tilt due to a high prevalence of false positive syncopal spells. In this patient population, a milder orthostatic stress was able to confirm CBF abnormalities in the absence of heart rate and blood pressure abnormalities, in patients with POTS and orthostatic hypotension.

## Data Availability

The datasets presented in this article are not readily available because they don't fulfill the rules where data availability is commonly needed to be made. Further enquiries can be directed to the corresponding author.
